# Plasma metabolomic signatures of migraine in 479,760 adults

**DOI:** 10.1016/j.isci.2026.117031

**Published:** 2026-08-06

**Authors:** Yanggang Hong, Feng Chen, Yi Wang, Xiu-Feng Huang

**Affiliations:** 1 The Second Affiliated Hospital and Yuying Children’s Hospital of Wenzhou Medical University, Wenzhou, Zhejiang, China; 2 Wenzhou Medical University, Wenzhou, Zhejiang, China

**Keywords:** migraine, metabolomics, plasma, lipoproteins, triglycerides

## Abstract

Migraine has been linked to metabolic and vascular factors, and previous metabolomic studies have implicated HDL-related alterations, but large-scale prospective evidence remains limited. We analyzed 251 baseline plasma metabolites measured by nuclear magnetic resonance in 479,760 UK Biobank participants, including 6,724 incident hospital-diagnosed migraine cases during follow-up. Prospective and cross-sectional analyses replicated and extended a coherent lipid and lipoprotein pattern: HDL-related measures were generally inversely associated with hospital-diagnosed migraine, whereas triglyceride-rich and VLDL-related measures showed positive associations. Repeated least absolute shrinkage and selection operator (LASSO) analysis prioritized 12 metabolite features, which were further examined in exploratory analyses of brain structural imaging phenotypes, migraine polygenic risk, and affective and sleep-related traits. These findings provide large-scale prospective evidence supporting a structured plasma metabolomic profile associated with hospital-diagnosed migraine and contextualize systemic lipid and lipoprotein metabolism as a relevant domain for future mechanistic and translational studies of migraine-related metabolic variation.

## Highlights

• Prospective plasma metabolomics identified metabolic signatures associated with migraine
• HDL-related measures were inversely associated with incident migraine
• Triglyceride-rich and VLDL measures showed positive associations with migraine
• LASSO prioritized 12 metabolites linked to brain, genetic, sleep, and affective traits

## Graphical abstract

**Figure:**
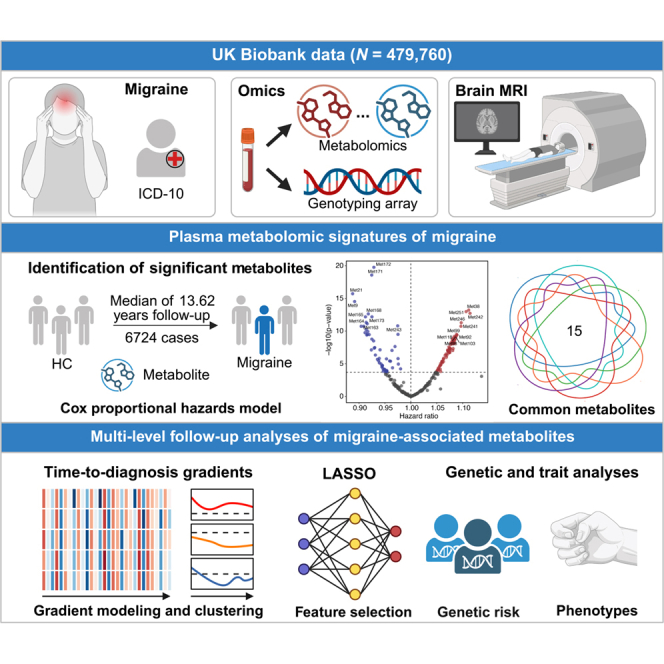

## Teaser

Health sciences; Medicine; Medical specialty; Internal medicine; Neurology; Public health; Biological sciences; Systems biology; Metabolomics

## Introduction

Migraine is a common and disabling neurological disorder characterized by recurrent headache attacks and a wide range of accompanying symptoms, including nausea, photophobia, phonophobia, and sensory hypersensitivity.^1^^,^^2^^,^^3^ It is one of the leading causes of years lived with disability worldwide, particularly among young and middle-aged adults, and imposes a substantial burden on individuals, healthcare systems, and society.^3^^,^^4^^,^^5^^,^^6^^,^^7^^,^^8^^,^^9^ Increasing evidence suggests that migraine is not merely an episodic headache condition, but a complex neurovascular disorder involving abnormal sensory processing, vascular dysregulation, cortical excitability, inflammation, and altered brain homeostasis.^10^^,^^11^^,^^12^^,^^13^^,^^14^^,^^15^^,^^16^^,^^17^^,^^18^ In addition, migraine frequently co-occurs with sleep disturbances, depression, anxiety, and other systemic conditions, highlighting its multifactorial and heterogeneous nature.^19^^,^^20^^,^^21^ Despite extensive investigation, the biological mechanisms underlying migraine onset and progression remain incompletely understood, and reliable biomarkers for risk stratification and early identification are still lacking.

Metabolomics has emerged as a powerful tool for capturing downstream molecular changes that reflect the integrated influence of genetic background, environmental exposures, lifestyle factors, and disease processes.^22^^,^^23^^,^^24^ By comprehensively profiling small molecules and metabolic intermediates in biological samples, metabolomics can provide insight into key physiological pathways, including lipid transport, fatty acid metabolism, amino acid metabolism, energy homeostasis, and inflammation.^25^^,^^26^^,^^27^^,^^28^ These pathways are biologically relevant to migraine, given previous evidence implicating mitochondrial dysfunction, oxidative stress, neuroinflammation, and vascular-metabolic imbalance in migraine pathophysiology. Several prior studies have reported altered metabolomic profiles in patients with migraine.^29^^,^^30^^,^^31^^,^^32^^,^^33^ However, many of these investigations were limited by relatively small sample sizes, case-control or cross-sectional designs, and restricted metabolite coverage. As a result, the prospective metabolomic architecture of migraine, particularly in large population-based settings, remains insufficiently characterized.

The UK Biobank (UKB) provides a unique platform for addressing these gaps. It is a large prospective population-based cohort with extensive phenotypic, biochemical, imaging, and genetic data collected under standardized protocols.^34^ In recent years, high-throughput nuclear magnetic resonance (NMR)-based metabolomics data have become available in UKB, enabling the quantification of a broad panel of circulating metabolic biomarkers, including detailed lipoprotein subclasses, cholesterol measures, fatty acids, amino acids, glycolysis-related metabolites, ketone bodies, and inflammatory markers.^35^^,^^36^ The scale and depth of UKB make it particularly well suited for investigating whether baseline plasma metabolic profiles are associated with future disease risk. Furthermore, the availability of brain magnetic resonance imaging (MRI) phenotypes, polygenic risk information, and a wide range of behavioral and psychological traits provides an opportunity to place migraine-associated metabolites into a broader neurobiological and genetic context.

In the present study, we systematically investigated the association between baseline plasma metabolites and migraine in the UKB. Specifically, we examined the relationships of 251 NMR-derived metabolic measures with incident and prevalent migraine, identified internally prioritized metabolite features with selection stability, and further explored their links with brain structural imaging phenotypes, migraine polygenic risk, and selected sleep-related and affective traits. By integrating these analyses, this study aimed to characterize the metabolic profile of migraine and provide new insights into its potential biological basis.

## Results

### Study population and metabolite profiling

A total of 479,760 participants without hospital-diagnosed migraine at baseline were included in the longitudinal analysis. During follow-up, 6,724 participants developed incident hospital-diagnosed migraine, whereas 473,036 had no incident hospital-diagnosed migraine. Baseline characteristics of the study population are presented in Table 1. Given the large sample size, standardized mean differences (SMDs) were used to describe the magnitude of between-group differences, while *p* values were provided for descriptive purposes. Participants who developed incident hospital-diagnosed migraine were more likely to be female and were slightly younger than those without incident hospital-diagnosed migraine. Most other baseline characteristics showed small between-group differences, with SMDs below 0.10 for BMI, ethnicity, educational attainment, smoking status, screen time, sleep duration, diabetes status, and cardiovascular disease status. In addition, a total of 251 baseline plasma metabolites measured using the Nightingale NMR platform were analyzed in this study. Metabolites were annotated according to predefined metabolite classes and lipoprotein subclasses to support module-level interpretation of lipid- and lipoprotein-related measures (Table S1).

**Table 1:** Baseline characteristics of participants included in the study

	Overall	Incident migraine	Healthy control	*p* value	SMD
***N***	479,760	6,724	473,036		
**Age (mean, SD)**	56.59 (8.10)	55.10 (8.47)	56.61 (8.10)	<0.001	0.182
**Sex,*****n*****(%)**				<0.001	0.368
Female	256,219 (53.4%)	4,756 (70.7%)	251,463 (53.2%)		
Male	223,541 (46.6%)	1,968 (29.3%)	221,573 (46.8%)		
**Ethnicity,*****n*****(%)**				0.116	0.029
White	452,503 (94.3%)	6,317 (93.9%)	446,186 (94.3%)		
Mixed	7,926 (1.7%)	120 (1.8%)	7,806 (1.7%)		
Asian or Asian British	11,206 (2.3%)	150 (2.2%)	11,056 (2.3%)		
Black or Black British	8,125 (1.7%)	137 (2.0%)	7,988 (1.7%)		
**Educational attainment (mean, SD)**	17.68 (2.66)	17.59 (2.69)	17.68 (2.66)	0.005	0.034
**BMI (mean, SD)**	27.45 (4.80)	27.55 (5.13)	27.45 (4.79)	0.091	0.020
**Townsend deprivation index (mean, SD)**	−1.28 (3.10)	−0.95 (3.24)	−1.29 (3.10)	<0.001	0.106
**Smoking status,*****n*****(%)**				0.064	0.029
Current	51,529 (10.7%)	679 (10.1%)	50,850 (10.7%)		
Previous	166,837 (34.8%)	2,295 (34.1%)	164,542 (34.8%)		
Never	261,394 (54.5%)	3,750 (55.8%)	257,644 (54.5%)		
**Alcohol consumption status,*****n*****(%)**				<0.001	0.144
Current	441,727 (92.1%)	5,906 (87.8%)	435,821 (92.1%)		
Previous	16,844 (3.5%)	375 (5.6%)	16,469 (3.5%)		
Never	21,189 (4.4%)	443 (6.6%)	20,746 (4.4%)		
**Television screen time (mean, SD)**	2.81 (1.68)	2.87 (1.73)	2.81 (1.68)	0.001	0.039
**Computer screen time (mean, SD)**	1.08 (1.39)	1.13 (1.48)	1.08 (1.39)	0.009	0.031
**Sleep duration (mean, SD)**	7.15 (1.11)	7.09 (1.26)	7.16 (1.11)	<0.001	0.056
**Diabetes,*****n*****(%)**	26,012 (5.4%)	324 (4.8%)	25,688 (5.4%)	0.030	0.028
**Cardiovascular disease,*****n*****(%)**	146,155 (30.5%)	2,057 (30.6%)	144,098 (30.5%)	0.829	0.003
**Follow-up years (mean, SD)**	13.19 (2.22)	7.42 (4.06)	13.27 (2.07)	<0.001	1.813

### Association between metabolites and incident migraine

**Figure 1:**
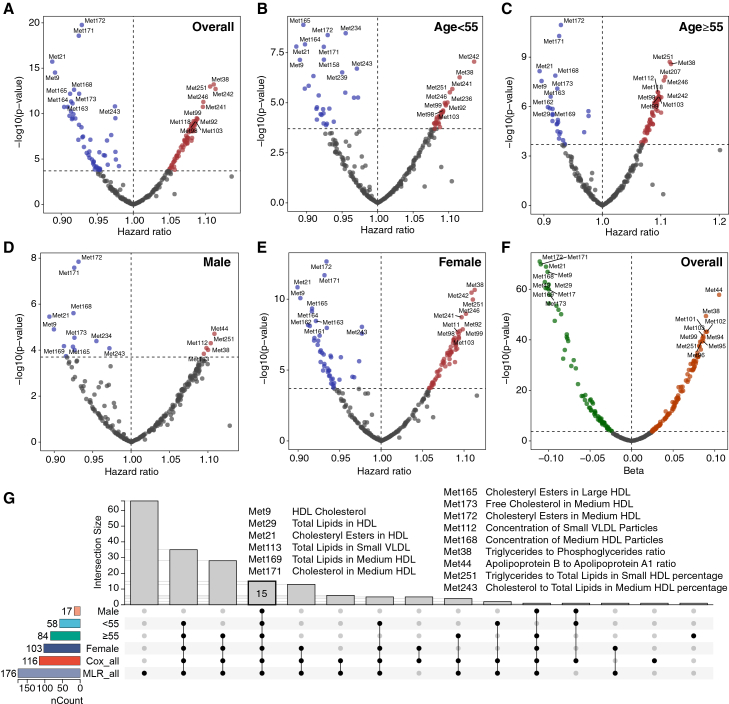
Plasma metabolites associated with migraine in overall, subgroup, and cross-sectional analyses (A–E) Volcano plots of the associations between baseline plasma metabolites and incident migraine in the overall population (A), participants aged <55 years (B), participants aged ≥55 years (C), men (D), and women (E), based on Cox proportional hazards models. The *x* axis shows the hazard ratio (HR) per standard deviation increase in metabolite level, and the *y* axis shows −log10(*p* value). The vertical dashed line indicates HR = 1, and the horizontal dashed line indicates the Bonferroni-corrected significance threshold. (F) Volcano plot of the cross-sectional associations between plasma metabolites and migraine in the overall population. The *x* axis shows the regression coefficient (beta), and the *y* axis shows −log10(*p* value). (G) UpSet plot summarizing the overlap of significant metabolites across longitudinal and cross-sectional analyses. Fifteen metabolites were consistently associated with migraine in both analytical frameworks with concordant directions of effect.

In the overall population, multiple baseline plasma metabolites were significantly associated with the risk of incident migraine (Figure 1A; Table S2). Both positive and inverse associations were observed. Metabolites related to HDL lipid composition were predominantly associated with a lower risk of incident migraine, whereas several triglyceride-rich and VLDL-related measures showed positive associations with future migraine risk. Among the most prominent inverse associations were HDL-related traits, including HDL cholesterol, total lipids in HDL, cholesteryl esters in HDL, cholesterol in medium HDL, and free cholesterol in medium HDL, while positive associations were observed for traits such as small VLDL particle concentration, triglycerides to phosphoglycerides ratio, and triglycerides to total lipids in small HDL percentage.

Age-stratified analyses showed that these associations were broadly directionally consistent across age groups, although the number and strength of significant associations differed between participants aged <55 years and those aged ≥55 years (Figures 1B and 1C; Tables S3 and S4). Similarly, sex-stratified analyses suggested an overall comparable metabolic pattern in men and women, with inverse associations mainly enriched in HDL-related measures and positive associations more evident for triglyceride- and lipoprotein-related metabolites (Figures 1D and 1E; Tables S5 and S6). However, the magnitude and statistical significance of individual metabolites varied between strata, indicating potential heterogeneity by age and sex.

To complement the longitudinal findings, cross-sectional analyses of prevalent migraine were performed. These analyses revealed a broadly similar pattern of association, with numerous metabolites demonstrating significant relationships with migraine status in the cross-sectional setting (Figure 1F; Table S7).

We further compared metabolite associations identified in the longitudinal Cox regression analyses, including the overall, age-stratified, and sex-stratified analyses, with those observed in the cross-sectional linear regression analysis. The UpSet plot identified 15 metabolites that overlapped across these analyses and showed consistent effect directions (Figure 1G; Table S8). These overlapping metabolites were primarily related to HDL lipid composition, medium HDL measures, small VLDL particles, and triglyceride-related ratios, supporting the robustness of the observed metabolic signatures across analytical frameworks.

Among the 15 overlapping metabolites, several HDL-related traits, including HDL cholesterol (Met9), total lipids in HDL (Met29), cholesteryl esters in HDL (Met21), concentration of medium HDL particles (Met168), cholesterol to total lipids in medium HDL percentage (Met243), cholesteryl esters in large HDL (Met165), total lipids in medium HDL (Met169), cholesterol in medium HDL (Met171), free cholesterol in medium HDL (Met173), and cholesteryl esters in medium HDL (Met172), were consistently inversely associated with migraine, whereas concentration of small VLDL particles (Met112), triglycerides to phosphoglycerides ratio (Met38), apolipoprotein B to apolipoprotein A1 ratio (Met44), triglycerides to total lipids in small HDL percentage (Met251), and total lipids in small VLDL (Met113) showed consistent positive associations. These findings indicate that perturbations in lipoprotein metabolism, particularly involving HDL- and triglyceride-related pathways, are strongly linked to migraine occurrence.

### Cross-sectional time-to-diagnosis gradients of migraine-associated metabolites

We next performed a matched case-control time-to-diagnosis gradient analysis to describe how baseline metabolite differences between future hospital-diagnosed migraine cases and matched controls varied according to the time interval between baseline assessment and subsequent diagnosis. For each matched set, metabolite levels were first residualized for covariates, and the case-control difference was then expressed as a standardized *Z* score relative to the matched controls. LOESS smoothing was used to visualize descriptive gradients across years before diagnosis.

**Figure 2:**
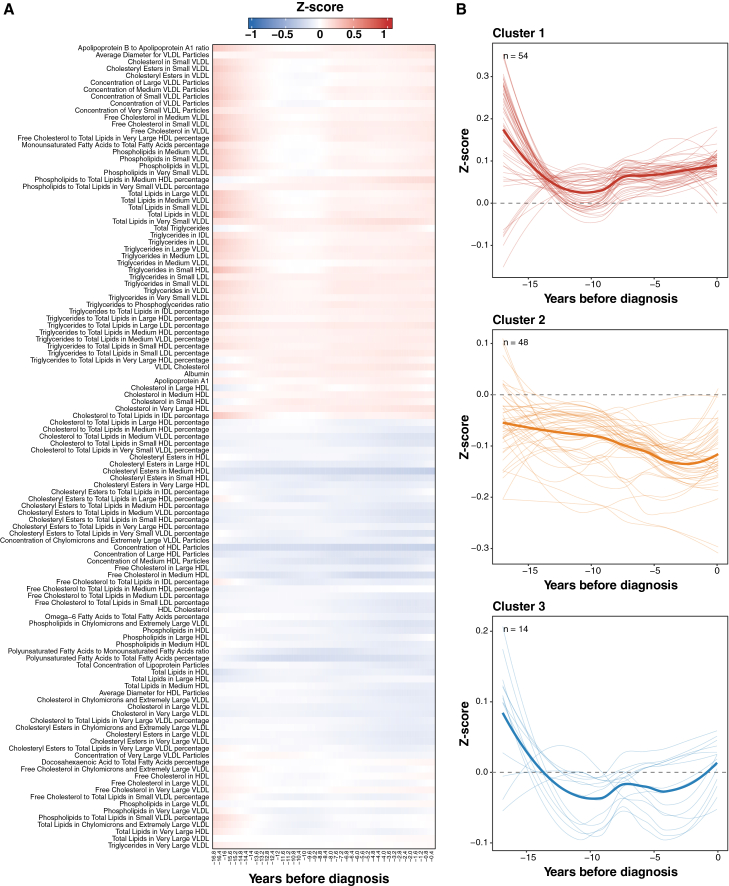
Cross-sectional time-to-diagnosis gradients of migraine-associated baseline metabolites (A) Heatmap showing LOESS-smoothed standardized case-control differences in baseline metabolite residuals across years before future hospital-diagnosed migraine. For each matched set, the residualized metabolite level of the incident migraine case was compared with the distribution of residualized metabolite levels among matched controls, yielding a standardized *Z* score. Positive values indicate higher baseline metabolite levels in future migraine cases relative to matched controls, whereas negative values indicate lower levels. (B) Descriptive clustering of LOESS-smoothed time-to-diagnosis gradients among migraine-associated metabolites. Thin lines represent individual metabolites, and thick lines represent the cluster-level mean pattern. Because plasma metabolites were measured only once at baseline, these curves represent between-person cross-sectional gradients aligned to future diagnosis time rather than within-person longitudinal trajectories.

The heatmap showed heterogeneous baseline case-control differences across migraine-associated metabolites. Triglyceride-rich and VLDL-related measures tended to show positive standardized differences in future migraine cases relative to matched controls, whereas several HDL-related cholesterol and lipid composition measures tended to show negative standardized differences (Figure 2A; Table S9). Descriptive clustering of the smoothed gradients identified three broad patterns (Figure 2B; Table S10). Cluster 1 (*n* = 54) showed an overall positive profile, cluster 2 (*n* = 48) showed a predominantly negative profile, and cluster 3 (*n* = 14) showed a more variable pattern across the time-to-diagnosis axis. This analysis provides a descriptive visualization of between-person baseline metabolite differences aligned to future hospital-diagnosed migraine timing. Because plasma metabolites were measured only once at baseline, these patterns should not be interpreted as within-person longitudinal trajectories or biologically distinct temporal subtypes.

### Metabolite feature prioritization

**Figure 3:**
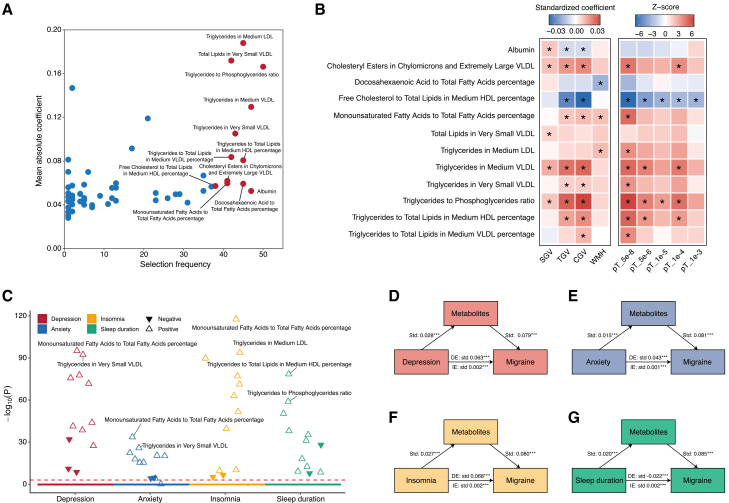
Multi-level follow-up analyses of prioritized migraine-associated metabolites (A) LASSO-based feature prioritization of candidate migraine-associated plasma metabolites. The *x* axis denotes selection frequency and the *y* axis denotes mean absolute coefficient. Red points indicate metabolites that met the predefined stability threshold and were retained as internally prioritized metabolite features, whereas blue points indicate non-selected metabolites. LASSO, least absolute shrinkage and selection operator. (B) Heatmaps showing the associations of prioritized metabolites with brain structural imaging phenotypes and migraine PRSs across multiple *p* value thresholds. Colors represent effect size magnitude and direction, and asterisks indicate statistical significance after Bonferroni correction. SGV, subcortical gray matter volume; TGV, total gray matter volume; CGV, cortical gray matter volume; WMH, white matter hyperintensity volume; PRS, polygenic risk score; PT, *p* value threshold. (C) Associations between prioritized metabolites and depression, anxiety, insomnia, and sleep duration. The y axis represents −log10(*p* value), with triangle direction indicating effect direction. The red horizontal dashed line indicates the Bonferroni-corrected significance threshold. (D–G) Exploratory structural equation models summarizing statistical interrelationships among depression (D), anxiety (E), insomnia (F), sleep duration (G), the latent metabolite factor, and incident hospital-diagnosed migraine. The latent metabolite factor was specified using the 12 LASSO-prioritized standardized metabolites as indicators. The values on arrows represent standardized model coefficients, and DE and IE denote model-based direct and indirect association parameters, respectively. These SEM results should not be interpreted as evidence of causal mediation. ∗*p* < 0.05, ∗∗*p* < 0.01, and ∗∗∗*p* < 0.001. DE, direct association parameter; IE, indirect association parameter.

To further prioritize metabolite features associated with incident hospital-diagnosed migraine and assess their internal selection stability, we performed repeated cross-validated least absolute shrinkage and selection operator (LASSO) logistic regression on the candidate metabolite set (Figure 3A; Table S11). This analysis was used as an internal feature-prioritization procedure rather than as a formal clinical prediction model or external biomarker validation framework.

Selection frequency varied substantially across metabolites, indicating different levels of stability in their contribution to the penalized logistic regression models across resampling iterations. Using a predefined stability threshold of selection in at least 75% of the 50 resampling iterations, 12 metabolites were retained as internally prioritized metabolite features. Among these features, triglycerides to phosphoglycerides ratio showed the highest selection frequency, being retained in all 50 iterations (100%) with a relatively large mean absolute coefficient (0.166). Other highly stable features included triglycerides in medium VLDL and albumin, each selected in 47 iterations (94%), as well as triglycerides in medium LDL, triglycerides to total lipids in medium HDL percentage, and docosahexaenoic acid to total fatty acids percentage, each selected in 45 iterations (90%). Additional metabolites meeting the stability criterion were triglycerides in very small VLDL, total lipids in very small VLDL, triglycerides to total lipids in medium VLDL percentage, cholesteryl esters in chylomicrons and extremely large VLDL, monounsaturated fatty acids to total fatty acids percentage, and free cholesterol to total lipids in medium HDL percentage, with selection frequencies ranging from 76% to 86%.

The magnitude of the mean absolute coefficients further highlighted several strongly weighted features in the internal prioritization model. Triglycerides in medium LDL showed the largest mean absolute coefficient (0.188), followed by total lipids in very small VLDL (0.172), triglycerides to phosphoglycerides ratio (0.166), triglycerides in medium VLDL (0.130), and triglycerides in very small VLDL (0.105). The prioritized features were predominantly enriched in triglyceride-related measures, VLDL/LDL lipid components, fatty acid composition traits, and albumin. These findings support the internal consistency of triglyceride-rich and lipoprotein-related metabolic signals within the present dataset. However, given the correlation structure of the NMR metabolomics panel, the reported selection frequencies should be interpreted as indicators of internal selection stability rather than evidence of independent or externally validated metabolite signals. Because external validation, calibration assessment, and incremental predictive performance analyses were not performed, these 12 metabolites should be interpreted as internally prioritized features associated with incident hospital-diagnosed migraine rather than as a validated biomarker panel for clinical risk prediction.

### Association between prioritized migraine-associated metabolites and brain structure

We next investigated whether the prioritized migraine-associated metabolites were related to structural brain imaging phenotypes in the imaging subset of the cohort. Several selected metabolites were significantly associated with brain structural measures after Bonferroni correction, with more evident signals observed for subcortical gray matter volume (SGV), total gray matter volume (TGV), and cortical gray matter volume (CGV) than for white matter hyperintensity (WMH) volume (Figure 3B; Table S12). Multiple triglyceride- and lipoprotein-related metabolites, including triglycerides in medium VLDL, triglycerides to phosphoglycerides ratio, and triglycerides to total lipids in medium HDL percentage, showed positive associations with gray matter volume phenotypes. By contrast, free cholesterol to total lipids in medium HDL percentage was inversely associated with TGV and CGV. Associations with WMH were more limited. Docosahexaenoic acid to total fatty acids percentage was inversely associated with WMH, whereas triglycerides in medium LDL measure was positively associated with WMH. Most other prioritized metabolites were not significantly related to this trait. These results indicate that the prioritized metabolite signature is linked not only to future migraine risk, but also to variation in structural brain imaging phenotypes, particularly gray matter volume measures.

### Integration with genetic risk and cross-trait associations

We next examined whether the prioritized migraine-associated metabolites were related to migraine polygenic risk score (PRS). Several selected metabolites were significantly associated with migraine PRS after Bonferroni correction across multiple PRS construction thresholds, although the strength and consistency of these associations varied across metabolites and thresholds (Figure 3B; Table S13). Triglyceride-related traits, VLDL-related measures, and fatty acid composition traits tended to show positive associations with migraine PRS, whereas free cholesterol to total lipids in medium HDL percentage showed an inverse association pattern. These findings suggest that part of the metabolite signature associated with incident hospital-diagnosed migraine may also be related to genetic liability to migraine.

We further assessed the associations between the selected metabolites and clinically relevant sleep-related and affective traits, including depression, anxiety, insomnia, and sleep duration. Multiple metabolites were significantly associated with these traits after Bonferroni correction, with stronger and more numerous signals observed for depression, insomnia, and sleep duration than for anxiety (Figure 3C; Table S14). In particular, monounsaturated fatty acids to total fatty acids percentage was associated with depression, anxiety, and insomnia. Triglycerides in very small VLDL was associated with depression and anxiety, whereas triglycerides in medium LDL was associated with insomnia. In addition, triglycerides to total lipids in medium HDL percentage and triglycerides to phosphoglycerides ratio were associated with sleep duration. These results suggest shared metabolic correlates across hospital-diagnosed migraine and related affective or sleep-related traits, but do not establish causal directionality.

To further summarize these interrelationships, exploratory structural equation models (SEMs) were fitted using a latent metabolite factor together with each sleep-related or affective trait and incident hospital-diagnosed migraine outcome (Figures 3D–3G). The latent metabolite factor was specified using the 12 LASSO-prioritized standardized metabolites as indicators with their loading structure reported (Table S15). Across the four models, each trait was associated with the latent metabolite factor, and the latent metabolite factor was associated with incident hospital-diagnosed migraine. The model-based indirect association parameters were statistically significant but small in magnitude. These SEM results should therefore be interpreted as exploratory statistical interrelationships rather than evidence of causal mediation. These integrative analyses suggest that the prioritized metabolite features are statistically connected with migraine PRS and with clinically relevant affective and sleep-related traits, while causal directionality remains unresolved.

## Discussion

In this large population-based study, we systematically characterized associations between baseline plasma metabolites and hospital-diagnosed migraine across multiple analytical dimensions. Several important findings emerged. First, multiple baseline circulating metabolites were associated with incident hospital-diagnosed migraine, with broadly similar patterns also observed in the prevalent migraine analysis. Second, the overlapping signals were mainly concentrated in lipid- and lipoprotein-related metabolic domains, with HDL-related measures generally showing inverse associations, whereas VLDL- and triglyceride-rich measures tended to show positive associations. Third, repeated cross-validated LASSO regression identified 12 internally prioritized metabolite features, many of which involved triglyceride metabolism, lipoprotein composition, fatty acid composition, and albumin. Finally, these prioritized metabolites were statistically related to brain structural imaging phenotypes, migraine PRSs, and affective and sleep-related traits, while exploratory structural equation modeling summarized statistical interrelationships among these traits, a latent metabolite factor, and incident hospital-diagnosed migraine. These findings support a coherent circulating lipid and lipoprotein metabolic pattern associated with hospital-diagnosed migraine.

Our findings should be interpreted in the context of previous metabolomic and lipoprotein studies of migraine. Onderwater et al. previously reported alterations in HDL metabolism in migraine using an NMR-based metabolomics platform,^30^ and Guo et al. further described phenotypic and genetic associations between migraine and lipoprotein subfractions.^37^ Therefore, the HDL-related associations observed in the present study should not be viewed as an entirely new discovery, but rather as a large-scale replication and extension of prior evidence. The main contribution of the present study is that it evaluates these metabolomic patterns in relation to both incident and prevalent hospital-diagnosed migraine in the UKB and demonstrates a coherent opposing lipoprotein pattern: HDL-related cholesterol, lipid, particle, and composition measures were generally inversely associated with hospital-diagnosed migraine, whereas triglyceride-rich and VLDL-related measures showed positive associations.

One of the most notable observations in our study was the contrast between HDL-related and triglyceride-rich/VLDL-related signals. Multiple HDL composition measures, particularly cholesterol- and lipid-related traits within HDL and medium HDL subclasses, were associated with lower risk of incident hospital-diagnosed migraine, whereas several VLDL- and triglyceride-dominant measures showed positive associations. This pattern is biologically plausible in light of the known roles of HDL particles in reverse cholesterol transport, anti-inflammatory activity, antioxidant functions, and endothelial-related processes.^38^^,^^39^ In contrast, triglyceride-rich lipoproteins are closely linked to metabolic dysfunction, vascular impairment, and low-grade systemic inflammation.^40^^,^^41^^,^^42^ Migraine has increasingly been studied as a neurovascular disorder with systemic components, and vascular reactivity, endothelial dysfunction, inflammatory activation, and altered energy balance have all been implicated in migraine-related pathophysiological processes.^43^ However, because the present study is observational, these associations should not be interpreted as evidence that HDL-related metabolites are protective or that triglyceride-rich lipoproteins causally increase migraine risk. Rather, they indicate that systemic lipid and lipoprotein metabolic status is associated with hospital-diagnosed migraine.

These results also fit with the broader concept that migraine may involve systemic metabolic correlates in addition to episodic headache generation. Previous studies have implicated mitochondrial dysfunction, oxidative stress, altered cerebral energy metabolism, and inflammatory signaling in migraine, while clinical and epidemiological studies have reported links between migraine and cardiometabolic traits.^44^^,^^45^^,^^46^ The predominance of triglyceride-related, VLDL-related, and fatty acid composition-related signals in the present study provides additional support for a metabolic dimension of hospital-diagnosed migraine. Albumin was also retained as one of the internally prioritized features in the LASSO analysis, which may be relevant given its roles in systemic nutritional and inflammatory status, as well as its potential association with vascular and metabolic homeostasis.^47^^,^^48^^,^^49^ Although the effect sizes of individual metabolites were modest, the overall pattern was coherent and biologically structured, suggesting that the findings are better interpreted at the level of lipid and lipoprotein metabolic domains rather than as isolated single-metabolite signals.

An important interpretational issue is the high correlation among many NMR-derived lipoprotein measures. Many Nightingale biomarkers are biologically and mathematically related because they quantify lipid concentrations, particle subclasses, and compositional percentages within shared lipoprotein systems. Therefore, the significant metabolites identified in this study should not be interpreted as numerous independent metabolic associations. Instead, our results suggest a module-level pattern, with inverse associations for HDL-related measures and positive associations for triglyceride-rich/VLDL-related measures. This interpretation is consistent with the structured nature of the NMR metabolomics platform and helps avoid overinterpretation of highly correlated individual biomarkers. Accordingly, the present findings are best understood as a coherent lipid and lipoprotein metabolic pattern associated with hospital-diagnosed migraine.

The matched time-to-diagnosis analysis was used to descriptively examine whether baseline metabolite differences between future hospital-diagnosed migraine cases and matched controls varied according to the interval between baseline assessment and subsequent diagnosis. Because plasma metabolites were measured only once at baseline, the fitted curves represent between-person cross-sectional gradients of baseline metabolite differences aligned to future diagnosis time rather than within-person longitudinal trajectories. Similarly, the clustering of LOESS-smoothed gradients was used only to summarize descriptive patterns in the baseline case-control differences and should not be interpreted as identifying biologically distinct temporal trajectory subtypes. Thus, this analysis provides a descriptive visualization of baseline metabolite differences across the time-to-diagnosis axis, but does not establish that metabolic perturbations evolve over time before migraine diagnosis.

The machine learning-based analysis further supported the internal consistency of the observed metabolic pattern. The LASSO-based analysis provided an additional internal feature-prioritization layer. Using repeated cross-validated LASSO logistic regression, we identified 12 metabolites that were repeatedly selected across resampling iterations. These features were largely dominated by triglyceride-rich lipoprotein traits, including triglycerides in medium LDL, medium VLDL, and very small VLDL, together with triglyceride-related ratio measures, selected fatty acid composition traits, and albumin. Given the strong correlation structure of the NMR metabolomics panel, particularly among lipoprotein-related measures, these 12 features should be interpreted as internally prioritized representatives of broader metabolic modules rather than as independent biological signals. This convergence between conventional regression and penalized feature prioritization supports the internal consistency of triglyceride-rich and VLDL-related signals within the present dataset. Similarly, the reported selection frequencies indicate internal selection stability within the present resampling framework, but do not establish causality, independence, external reproducibility, or clinical predictive utility. However, the LASSO framework was not intended to develop a clinical prediction model, and the selected features were not externally validated. Therefore, these metabolites should be described as internally prioritized metabolite features associated with incident hospital-diagnosed migraine rather than as a reproducible biomarker panel or clinically validated prediction model. External validation in independent cohorts with comparable metabolomic profiling remains necessary before these features can be considered reproducible biomarkers or used for clinical risk prediction.^50^^,^^51^

We further contextualized the prioritized metabolites by examining their associations with brain structural MRI phenotypes. Several metabolites showed stronger associations with gray matter-related measures than with WMH burden, suggesting that circulating metabolic variation associated with hospital-diagnosed migraine may also be statistically related to structural brain characteristics. This observation is broadly consistent with prior literature suggesting that migraine may be associated with altered brain morphology and network-level brain changes, although previous findings have been heterogeneous.^52^^,^^53^^,^^54^^,^^55^ Nevertheless, because brain MRI data were acquired at dedicated imaging visits after baseline and at varying times relative to migraine onset, these findings should not be interpreted causally or directionally. Rather, they indicate that metabolite features associated with hospital-diagnosed migraine may also correlate with neurostructural phenotypes.

Another aspect of this study is the integration of metabolite findings with inherited genetic liability and cross-trait associations. Several prioritized metabolites were associated with migraine PRSs across multiple thresholds, suggesting that part of the metabolomic pattern associated with hospital-diagnosed migraine may be statistically related to genetic liability to migraine.^37^ In addition, many selected metabolites were associated with depression, anxiety, insomnia, and sleep duration, traits that frequently co-occur with migraine in clinical and population settings.^56^ These findings suggest shared metabolic correlates across hospital-diagnosed migraine and affective or sleep-related traits, but they do not establish causal directionality. Consistent with this cautious interpretation, the exploratory structural equation models were used only to summarize statistical interrelationships among these traits, the latent metabolite factor, and incident hospital-diagnosed migraine. Although model-based direct and indirect association parameters were estimated, these parameters should not be interpreted as evidence of formal causal mediation, because temporal ordering among all variables could not be fully established and residual confounding cannot be excluded.

The interpretation of these findings should be anchored in the migraine phenotype used in this study. Hospital inpatient ICD-10 G43 records were used to define hospital-diagnosed migraine, providing a date-resolved and clinically specific phenotype suitable for prospective time-to-event analyses. This design prioritizes diagnostic specificity and temporal alignment between baseline metabolomics and subsequent recorded migraine diagnosis, but does not capture the full community spectrum of migraine, including milder or primary-care-managed cases. Future studies integrating self-report, primary-care records, outpatient diagnoses, and prescription-based information will be important to determine whether the lipid and lipoprotein pattern observed here generalizes across migraine severity levels and healthcare pathways. Because hospital records have limited sensitivity, some participants classified as non-cases may have had unrecorded migraine. Such outcome under-ascertainment and reference-group contamination would generally be expected to attenuate associations toward the null rather than create a coherent opposing lipoprotein pattern. Nevertheless, the magnitude and generalizability of these associations require validation in cohorts with broader and more granular migraine ascertainment.

Because the central findings involved lipid and lipoprotein measures, potential confounding by lipid-lowering medication use should also be considered. Statins and other lipid-lowering therapies may directly influence circulating lipoprotein concentrations and could therefore affect the observed metabolomic associations. Time-updated medication changes during follow-up could not be reliably modeled in the full UKB metabolomics cohort. Therefore, we did not interpret these associations as medication-independent or causal metabolic signatures. In addition, hospital-diagnosed migraine may partly reflect healthcare-contact intensity, because participants with more frequent healthcare contact may be more likely to receive migraine diagnoses, cardiometabolic assessment, and lipid-lowering treatment. Future studies with more complete longitudinal medication data and healthcare-utilization measures are needed to evaluate whether these lipid and lipoprotein associations persist independently of treatment patterns and healthcare-contact intensity.

The present study has several strengths. It was conducted in a large, well-characterized population-based cohort with standardized metabolomic profiling and prospective follow-up, allowing us to examine baseline metabolites in relation to incident hospital-diagnosed migraine. We analyzed a broad panel of 251 NMR-derived plasma metabolites and combined multiple complementary strategies, including Cox regression, prevalent migraine analysis, matched cross-sectional time-to-diagnosis gradient analysis, internal feature prioritization, neuroimaging integration, PRS analysis, and exploratory SEM. This multidimensional design enabled us to identify coherent metabolite patterns associated with hospital-diagnosed migraine and to contextualize them across imaging, genetic, and behavioral domains. Importantly, these downstream analyses were used to provide exploratory context rather than to establish causal mechanisms.

In conclusion, this study provides large-scale prospective evidence supporting and extending a coherent plasma metabolomic pattern associated with hospital-diagnosed migraine, characterized predominantly by inverse associations for HDL-related traits and positive associations for triglyceride-rich/VLDL-related measures. These findings reinforce and extend prior evidence linking migraine with HDL metabolism and lipoprotein subfractions. The internally prioritized metabolite features were further statistically connected with brain structural phenotypes, migraine PRSs, and affective and sleep-related traits. Overall, our results support the relevance of systemic lipid and lipoprotein metabolism in hospital-diagnosed migraine, while emphasizing that the observed associations do not establish causality, within-person longitudinal metabolic evolution, or a clinically validated prediction model.

### Limitations of the study

Several limitations should be acknowledged. First, migraine was ascertained using hospital inpatient ICD-10 records and should therefore be interpreted as hospital-diagnosed migraine. Although this approach provides a clinically specific phenotype, it likely has limited sensitivity for migraine in the general population and may preferentially capture more severe, complicated, or healthcare-contact-intensive cases, while some participants classified as non-cases may have had unrecorded migraine. Second, many NMR-derived lipoprotein measures are biologically and mathematically correlated. Thus, the findings should be interpreted as module-level lipid and lipoprotein patterns, mainly involving HDL-related and triglyceride-rich/VLDL-related domains, rather than as numerous independent metabolite associations. Third, residual confounding cannot be excluded. Dietary intake, physical activity, lipid-lowering medication use, changes in lipid-lowering therapy during follow-up, and healthcare-utilization patterns were not fully captured or modeled, which may have influenced the observed lipid and lipoprotein associations. Fourth, plasma metabolites were measured only once at baseline, so the time-to-diagnosis gradient analyses reflect descriptive between-person cross-sectional patterns aligned to future diagnosis time rather than within-person longitudinal metabolic trajectories. Fifth, the MRI analyses were cross-sectional with non-uniform temporal ordering relative to migraine onset, and the PRS and SEM analyses were exploratory. Therefore, these analyses should not be interpreted as evidence of causal mechanisms or formal mediation. Finally, UKB is not fully representative of the general population and predominantly includes individuals of European ancestry, and the internally prioritized metabolite features have not been validated in an independent external cohort.

## Resource availability

### Lead contact

Requests for further information and resources should be directed to and will be fulfilled by the lead contact, Yanggang Hong (sun160414@icloud.com).

### Materials availability

This study did not generate new unique reagents.

### Data and code availability

• Individual-level UKB data used in this study are available to approved researchers through the UKB application system and cannot be publicly redistributed by the authors because of data-access restrictions. Detailed information on UKB data access is available through the UKB research access platform (https://www.ukbiobank.ac.uk/).
• No new software was developed in this study. The analysis code used in this study is publicly available at https://github.com/sun160414/UKB_Metabolomic_Migraine.
• All supplementary tables generated in this study are provided with the manuscript. Any additional information required to reanalyze the data reported in this paper is available from the lead contact upon reasonable request.

## Acknowledgments

We thank the participants and investigators of UKB for generating and providing access to the data used in this study. This research was conducted using the UKB Resource under approved application number 1260511.

## Author contributions

Y.H. conceptualized and designed the study; supervised the research; administered the project; conducted the investigation, validation, and visualization; drafted the initial manuscript; and reviewed and edited the manuscript. F.C. contributed to investigation, validation, and manuscript review and editing. Y.W. contributed to validation and manuscript review and editing. X.-F.H. contributed to manuscript review and editing. All authors reviewed, revised, and approved the final version of the manuscript.

## Declaration of interests

The authors declare no competing interests.

## Declaration of generative AI and AI-assisted technologies in the writing process

During the preparation of this work, the authors used ChatGPT in order to assist with language editing and text refinement. After using this tool, the authors reviewed and edited the content as needed and take full responsibility for the content of the publication.

## STAR★Methods

### Key resources table

**Table:** 

REAGENT or RESOURCE	SOURCE	IDENTIFIER
**Deposited data**		

UK Biobank data	UK Biobank	https://www.ukbiobank.ac.uk/
GWAS summary statistics	FinnGen	https://www.finngen.fi/en

**Software and algorithms**		

R v.4.2.1	R Foundation	https://www.r-project.org/
Python v.3.11.11	Python Software Foundation	https://www.python.org/
mice v.3.19.0	CRAN	https://cran.r-project.org/package=mice
MatchIt v.4.7.2	CRAN	https://cran.r-project.org/package=MatchIt
survival v.3.8–6	CRAN	https://cran.r-project.org/package=survival
lavaan v.0.6–21	CRAN	https://cran.r-project.org/package=lavaan
PRSice-2	Choi SW et al.^57^	https://www.prsice.info/
scikit-learn v.1.8.0	scikit-learn developers	https://scikit-learn.org/

**Other**		

Code	This paper	https://github.com/sun160414/UKB_Metabolomic_Migraine

### Experimental model and study participant details

#### Human subject characteristics

##### Ethics

The study was conducted in accordance with the Declaration of Helsinki. UKB received ethical approval from the North West Multi-centre Research Ethics Committee (21/NW/0157), and this research was conducted under approved UKB application number 1260511. Publicly available summary-level GWAS data from FinnGen were de-identified and generated with appropriate local ethical approval and participant consent.

##### Study population and design

This study was conducted using data from the UKB, a large prospective population-based cohort of approximately 500,000 participants aged 40–69 years recruited between 2006 and 2010.^34^ Baseline data included questionnaire information, physical measurements, and biological samples. Plasma metabolomic measurements were available for a subset of participants at baseline. For incident analyses, individuals with migraine before baseline were excluded. Participants were followed from baseline until first migraine diagnosis, death, loss to follow-up, or end of follow-up, whichever occurred first.

### Method details

#### Plasma metabolomics profiling

Plasma metabolomic profiling was performed at baseline using the Nightingale Health high-throughput nuclear magnetic resonance (NMR) biomarker platform implemented in the UKB.^36^^,^^58^^,^^59^ A total of 251 metabolic measures were included in this study, comprising absolute concentrations and predefined biologically informative ratio or percentage measures. These metabolites covered major metabolic categories, including amino acids, apolipoproteins, cholesterol, fatty acids, glycolysis-related metabolites, inflammation-related biomarkers, ketone bodies, lipoproteins, and lipids.^28^ To improve biological interpretability and account for the structured correlation among NMR-derived lipoprotein biomarkers, all metabolites were annotated according to predefined metabolite classes and lipoprotein subclasses (Table S1). These annotations were used to support module-level interpretation of the findings, particularly for HDL-, LDL-, VLDL-, IDL-, triglyceride-, cholesterol-, and phospholipid-related measures. After sample-level quality control, missing metabolite values were imputed using metabolite-specific medians. Metabolite values were natural log-transformed to reduce skewness and then standardized to z-scores before analysis. Detailed information on sample handling, platform-level quality control, metabolite annotation, and preprocessing is provided in Methods S1.

#### Assessment of migraine status

Migraine status was ascertained using linked UKB electronic health records, primarily hospital inpatient records. Hospital-diagnosed migraine was defined using the ICD-10 code G43. The earliest recorded hospital diagnosis date was used as the event date. Prevalent hospital-diagnosed migraine was defined as any G43 diagnosis recorded on or before baseline, whereas incident hospital-diagnosed migraine was defined as a first-ever G43 diagnosis after baseline among participants without hospital-diagnosed migraine at baseline. This hospital-based definition was intended to capture a clinically recognized and high-specificity migraine phenotype, although it may not represent the full spectrum of migraine managed in outpatient or community settings. Detailed information on migraine ascertainment and phenotype definition is provided in Methods S1.

#### Covariates

Covariates were selected *a priori* based on their potential associations with migraine and metabolomic variation. These included age, sex, ethnicity, Townsend deprivation index, educational attainment, body mass index, smoking status, alcohol consumption, screen time, sleep duration, diabetes, cardiovascular disease, and medication for cholesterol, blood pressure, or diabetes. All covariates were defined at baseline. Missing covariate data were handled using multiple imputation by chained equations. Detailed variable definitions and coding are provided in Methods S1.

### Quantification and statistical analysis

#### Association analyses of plasma metabolites with migraine

Associations between baseline plasma metabolites and incident migraine were evaluated using Cox proportional hazards regression models, with follow-up time since baseline used as the timescale. Hazard ratios (HRs) and 95% confidence intervals (CIs) were estimated per 1-standard deviation increase in metabolite level. Cross-sectional associations with prevalent migraine were examined using multivariable linear regression. All models were adjusted for the covariates described above. Exploratory stratified analyses were further performed by sex and age group. The proportional hazards assumption was assessed using Schoenfeld residual-based diagnostics, and no major violations were identified in the primary Cox models. Multiple testing was controlled using Benjamini–Hochberg false discovery rate (FDR) correction and Bonferroni correction. Additional details regarding model specification, subgroup analyses, and diagnostic procedures are provided in Methods S1.

#### Downstream integrative analyses

To further characterize metabolite patterns associated with hospital-diagnosed migraine, we conducted several downstream analyses. A matched cross-sectional time-to-diagnosis gradient analysis was used to describe baseline metabolite differences between future incident migraine cases and matched controls according to the interval from baseline assessment to subsequent diagnosis. Repeated cross-validated LASSO logistic regression was used to identify 12 internally prioritized metabolite features. Their associations with brain structural MRI phenotypes, migraine PRSs, and selected sleep-related and affective traits were further evaluated using multivariable regression models, with Bonferroni correction applied according to the number of tests within each downstream analysis. Finally, exploratory SEM was used to summarize statistical interrelationships among selected sleep-related or affective traits, a latent metabolite factor based on the 12 LASSO-prioritized metabolites, and incident hospital-diagnosed migraine. These SEM analyses were interpreted as exploratory statistical associations rather than evidence of formal causal mediation. Detailed procedures for the time-to-diagnosis gradient analysis, LASSO analysis, MRI analyses, PRS construction, cross-trait association analyses, and exploratory SEM are described in Methods S1.

## Supplemental information

Methods S1. Detailed methods for plasma metabolomics profiling, migraine ascertainment, covariate handling, and downstream integrative analyses

Table S1. Detailed information on the plasma metabolites included in the study

Table S2. Associations between plasma metabolites and incident migraine in the overall population

Table S3. Associations between plasma metabolites and incident migraine in participants aged <55 years

Table S4. Associations between plasma metabolites and incident migraine in participants aged ≥55 years

Table S5. Associations between plasma metabolites and incident migraine in male participants

Table S6. Associations between plasma metabolites and incident migraine in female participants

Table S7. Cross-sectional associations between migraine and plasma metabolites

Table S8. Overall association estimates for the 15 overlapping plasma metabolites identified in both longitudinal and cross-sectional analyses with consistent effect directions

Table S9. Cross-sectional time-to-diagnosis gradients of migraine-associated baseline metabolites

Table S10. Clusters of cross-sectional time-to-diagnosis gradients among migraine-associated metabolites

Table S11. LASSO-based feature selection results for plasma metabolites associated with incident migraine

Table S12. Associations between migraine-associated plasma metabolites and brain structural imaging phenotypes

Table S13. Associations between migraine-associated plasma metabolites and genetic risk of migraine across five *p* value thresholds

Table S14. Associations between migraine-associated plasma metabolites and potential explanatory factors of migraine

Table S15. Standardized factor loadings of the latent metabolite factor in exploratory structural equation models
