# Supplementary material for: Plasma metabolomic signatures of migraine in 479,760 adults
Source: iScience. 2026 Aug 6;29(8):117031. doi: 10.1016/j.isci.2026.117031 (PMC13475662; doi:10.1016/j.isci.2026.117031)
Supplement: Supplementary file 1 [file mmc1.pdf]

iScience, Volume *29*

Supplemental information

Plasma metabolomic signatures of migraine in 479,760 adults

Yanggang Hong, Feng Chen, Yi Wang, and Xiu-Feng Huang

METHODS S1: Detailed methods for plasma metabolomics profiling, migraine ascertainment, covariate handling, and downstream integrative analyses

1. Ethics

This study was conducted in accordance with the principles of the Declaration of Helsinki. Ethical approval for the UK Biobank (UKB) was granted by the North West Multi-centre Research Ethics Committee, and all participants provided written informed consent at recruitment (ethics approval number: 21/NW/0157). All analyses involving individual-level UKB data were conducted under approved UKB applications in accordance with the UKB access policies and data governance requirements. This research was conducted using the UKB Resource under approved application number 1260511. Summary-level genome-wide association study (GWAS) data used for polygenic risk score analyses were obtained from FinnGen and were publicly available, de-identified, and generated with appropriate local ethical approval and participant consent.

2. Study population and design

The present study was conducted within the UKB, a large prospective population-based cohort that recruited approximately 500,000 participants aged 40–69 years across the United Kingdom between 2006 and 2010.[1] Baseline assessments included touchscreen questionnaires, verbal interviews, physical measurements, and collection of biological samples. Plasma metabolomic profiling was available for a subset of participants at baseline through the Nightingale Health nuclear magnetic resonance (NMR) platform.

For the primary prospective analyses, participants with evidence of migraine prior to or at baseline were excluded to ensure an incident disease design. Follow-up time was calculated from the date of baseline assessment to the earliest of the following events: first recorded hospital diagnosis of migraine, death, loss to follow-up, or end of follow-up. Participants free of migraine at baseline contributed person-time until censoring or incident migraine onset. Separate cross-sectional analyses of prevalent migraine were performed at baseline using the same metabolomic measurements and covariates.

3. Plasma metabolomics profiling and preprocessing

Blood samples were collected in ethylenediaminetetraacetic acid (EDTA) vacutainers and were promptly centrifuged at 2,500 × g for 10 min at 4 °C to separate plasma, which was subsequently stored at −80 °C until analysis. Detailed procedures for blood collection, handling, and long-term storage have been described previously.[2] Metabolic profiling of EDTA plasma samples was performed using the Nightingale Health high-throughput NMR biomarker platform, as implemented in the UKB.[3-5] This platform quantifies a broad panel of circulating metabolic biomarkers, including routine lipids, lipoprotein subclass composition measures, fatty acids, amino acids, glycolysis-related metabolites, ketone bodies, apolipoproteins, and inflammatory biomarkers. Detailed information on platform methodology, sample preparation, biomarker quantification, and platform-level quality control is publicly available in the UKB NMR companion documentation.

A total of 251 metabolic measures were included in the present study. According to the UKB NMR classification framework, these measures comprised both absolute metabolite concentrations and predefined biologically informative ratio or percentage measures derived through knowledge-based integration of existing biomarkers. These biomarkers were grouped into 10 major categories, including amino acids, apolipoproteins, cholesterol, fatty acids, fluid balance, glycolysis-related metabolites, inflammation-related biomarkers, ketone bodies, lipoproteins, and lipids.[6] Predefined ratio measures generated by the Nightingale platform, such as apolipoprotein B/apolipoprotein A1, omega-6/omega-

3 fatty acids, and polyunsaturated fatty acids/monounsaturated fatty acids, were retained in the analysis.

To improve biological interpretability in the context of the strong correlation structure of NMR-derived lipoprotein biomarkers, all 251 metabolites were further annotated according to predefined metabolite classes and lipoprotein subclasses. Annotation categories included major metabolite class, lipoprotein particle class, lipoprotein subclass or particle size, molecular component, and whether the biomarker represented an absolute measure, ratio, or percentage. This annotation was used to support module-level interpretation of the findings, particularly for highly correlated HDL-, LDL-, VLDL-, IDL-, triglyceride-, cholesterol-, and phospholipid-related measures. The metabolite annotation table is provided in Table S1.

For preprocessing, NMR biomarker variables measured at the baseline assessment were extracted using the corresponding UKB field identifiers. Sample-level quality control was conducted using the Nightingale NMR quality flag provided by UKB. Participants with failed sample quality control were excluded, whereas individuals with missing quality flag values were retained. Metabolite variables were then screened for missingness, and missing values were imputed using the median value of each metabolite. To reduce skewness and improve comparability across biomarkers, all metabolite measures included in the analysis were natural log-transformed. For variables containing non-positive values, values less than or equal to zero were replaced with half of the smallest positive value observed for that metabolite before log transformation. If no positive value was available for a given metabolite, the transformed value was set to missing. After log transformation, all metabolite measures were standardized to standard deviation units using z-score normalization before downstream analyses. The log transformation was used to reduce skewness and the influence of extreme values, rather than to require strict normality of all metabolite predictors. Cox regression does not require predictor variables to follow a normal distribution; nevertheless, log transformation and z-score standardization improved the comparability and numerical stability of metabolite-level association analyses.

4. Assessment of migraine status

Migraine status was ascertained using linked electronic health records in the UKB, with a primary focus on hospital inpatient data. Throughout this study, the outcome was therefore defined as hospital-diagnosed migraine. Cases were identified using the International Classification of Diseases, Tenth Revision (ICD-10) code G43. In UKB inpatient data, ICD-10 diagnosis codes are available under field ID 41270, and the corresponding diagnosis dates are available under field ID 41280 across inpatient episodes.

The event date for migraine was defined as the earliest available hospital diagnosis date for ICD-10 code G43 across all eligible inpatient records. Prevalent hospital-diagnosed migraine was defined as any recorded G43 diagnosis occurring prior to or on the date of baseline assessment. Incident hospital-diagnosed migraine was defined as a first-ever G43 diagnosis recorded after baseline among participants without evidence of prior hospital-diagnosed migraine at or before baseline.

This definition was intended to capture a clinically recognized and high-specificity migraine phenotype recorded in hospital settings. However, we acknowledge that hospital inpatient records are unlikely to capture the full spectrum of migraine in the general population, particularly milder or less clinically complicated cases managed exclusively in primary care, outpatient clinics, or without formal medical contact. Therefore, this phenotype may be enriched for more severe, complicated, or healthcare-contact-intensive migraine presentations. In addition, some participants classified as non-cases may have had unrecorded migraine, which could introduce outcome misclassification.

Accordingly, the findings should be interpreted as associations with hospital-diagnosed migraine rather than with migraine of all severities, and their generalizability to the broader migraine population should be considered with caution.

5. Covariate assessment and handling

Covariates were selected a priori on the basis of established associations with migraine risk and their potential to confound circulating metabolomic measurements. Demographic variables included age at baseline (field 21022), sex (field 31), and ethnicity (field 21000), categorized according to standard UKB classifications. Socioeconomic status was assessed using the Townsend deprivation index (TDI; field 22189), treated as a continuous variable, with higher values indicating greater material deprivation. Educational attainment was derived from baseline questionnaire data (fields 845, 6138, and 6142) and harmonized into ordered categories or years of education as appropriate.

Lifestyle-related covariates included body mass index (BMI; field 21001), smoking status (field 20116), and alcohol consumption status (field 20117), all assessed at baseline. Additional phenotype-related covariates were incorporated to capture behavioral and cardiometabolic factors potentially relevant to both migraine susceptibility and metabolic variation. These included daily screen time spent watching television (field 1070) and using a computer (field 1080), average sleep duration (field 1160), diabetes status (field 2443), cardiovascular disease (CVD) status (field 6150), and medication for cholesterol, blood pressure, or diabetes (field 6177).

All covariates were defined using baseline information and treated as fixed covariates in regression analyses unless otherwise specified. Categorical variables were modeled using indicator variables where appropriate. Missing values in covariates were imputed using multiple imputation by chained equations (MICE) implemented in the R package mice (v3.19.0) under R (v4.2.1), with variable-specific imputation models selected according to data type. Imputed covariates were handled consistently across primary, stratified, and downstream analyses.

6. Association analyses of plasma metabolites with incident and prevalent migraine

Time-to-event analyses were performed to evaluate the associations between baseline plasma metabolites and the risk of incident migraine. Participants with migraine prior to baseline were excluded from all survival analyses. For each metabolite, Cox proportional hazards regression models were fitted using follow-up time since baseline as the time scale to estimate hazard ratios (HRs) and 95% confidence intervals (CIs) per 1-SD increase in metabolite level. Multivariable models were adjusted for the covariates described above.

To explore the consistency of associations across demographic strata, exploratory subgroup analyses were additionally conducted according to sex (male and female) and age at baseline (<55 years and ≥55 years). Within each stratum, the same modeling framework and covariate adjustment strategy were applied, with the stratifying variable omitted from the corresponding model. Subgroup findings were interpreted cautiously because of reduced case numbers and corresponding reductions in statistical power. The proportional hazards assumption was assessed using Schoenfeld residual-based diagnostics, and no major violations were identified in the primary Cox models.

Cross-sectional analyses of prevalent migraine were conducted using multivariable linear regression models, with standardized metabolite levels modeled as dependent variables and prevalent migraine status treated as the exposure variable. Effect estimates were reported as regression coefficients (β) with 95% CIs, representing the mean difference in standardized metabolite levels associated with prevalent migraine. The same set of covariates was used as in the prospective analyses.

To account for multiple testing across the 251 metabolic measures, Benjamini–Hochberg false

discovery rate (FDR) correction and Bonferroni correction were applied. Bonferroni correction was used as a conservative threshold and was not intended to imply statistical independence among the highly correlated Nightingale NMR biomarkers. Given the known correlation structure of lipoprotein-related measures, significant findings were interpreted primarily at the level of predefined metabolite classes and lipoprotein subclasses rather than as fully independent single-metabolite associations. All survival and regression analyses were performed in R (v4.2.1).

7. Matched cross-sectional time-to-diagnosis gradient analysis and clustering

To descriptively characterize baseline metabolite differences according to the timing of future hospital-diagnosed migraine, we performed a matched cross-sectional time-to-diagnosis gradient analysis among participants without hospital-diagnosed migraine at baseline. This design follows the logic of incidence-density sampling.[7] Incident hospital-diagnosed migraine cases were matched to controls using the R package MatchIt (v4.7.2), with nearest-neighbor matching based on Mahalanobis distance and exact matching on sex.[8] Cases were matched to controls at a ratio of 1:10. Matching variables included the baseline covariates described above, including age, ethnicity, educational attainment, Townsend deprivation index, body mass index, smoking status, alcohol consumption, screen time, sleep duration, diabetes status, and cardiovascular disease status. Matched datasets were extracted using match.data, and covariate balance was assessed using standard MatchIt diagnostics. A time-to-diagnosis axis was created within each matched subclass. For each matched set, the diagnosis time of the incident migraine case was assigned to the corresponding matched set and multiplied by −1 to represent years before diagnosis. This procedure aligned baseline metabolite measurements from future migraine cases and matched controls according to the future diagnosis time of the case.

To reduce the influence of measured covariates on metabolite levels, each metabolite was first residualized in the matched dataset by fitting a linear regression model adjusted for the same baseline covariates. Residualized metabolite values were retained at the individual level. Within each matched subclass, the residualized metabolite level of the incident migraine case was then compared with the distribution of residualized metabolite levels among matched controls. This case-control difference was expressed as a standardized Z-score, calculated as the case residual minus the mean control residual divided by the standard deviation of control residuals within the same matched subclass. When the standard deviation among controls within a subclass was zero, the corresponding standardized value was set to missing.

For each metabolite, locally estimated scatterplot smoothing (LOESS) was applied to visualize the relationship between subclass-level standardized case-control differences and years before diagnosis.[7,9,10] LOESS-predicted values were generated on a common time grid to facilitate comparison across metabolites. This analysis was restricted to metabolites identified as significant in the primary association analyses. Hierarchical clustering was performed on the LOESS-smoothed time-to-diagnosis gradient matrix using Euclidean distance and Ward's minimum variance method. Metabolites were classified into three descriptive gradient clusters. Cluster patterns were visualized using heatmaps of LOESS-smoothed standardized values across the time-to-diagnosis axis, together with metabolite-level gradient plots and cluster-wise mean patterns.

Importantly, because plasma metabolites were measured only once at baseline, this analysis does not estimate within-person longitudinal metabolic changes. The resulting curves should therefore be interpreted as descriptive between-person cross-sectional gradients of baseline metabolite differences aligned to future hospital-diagnosed migraine timing, rather than true pre-diagnostic metabolic trajectories.

8. Internal prioritization of metabolite features using LASSO logistic regression

To prioritize metabolites associated with incident hospital-diagnosed migraine status and assess their internal selection stability, we applied a repeated cross-validation framework based on least absolute shrinkage and selection operator (LASSO)-penalized logistic regression.[11] In this analysis, incident hospital-diagnosed migraine was treated as a binary outcome indicating whether a participant developed a first hospital-recorded ICD-10 G43 diagnosis during follow-up. The time-to-event structure and censoring information were addressed in the primary Cox proportional hazards models; therefore, the LASSO analysis was used only as an internal feature-prioritization and selection-stability assessment procedure.

The candidate predictor set consisted of preprocessed and standardized plasma metabolite variables. Sample identifiers and outcome variables were excluded before model fitting. Because the analytical matrix contained no missing values after preprocessing, no additional imputation was required at this stage. All predictors were standardized within the modeling pipeline using z-score transformation.

Feature selection was conducted using repeated stratified five-fold cross-validation with 10 repeats, yielding 50 resampling iterations. In each iteration, an L1-penalized logistic regression model was fitted using the liblinear solver, with the regularization parameter set to C = 0.1 and the maximum number of iterations set to 5,000. Model discrimination within each held-out test fold was summarized using the area under the receiver operating characteristic curve (AUC). These AUC values were used only to describe internal model performance during resampling and were not interpreted as evidence of clinical predictive utility.

Metabolites with non-zero coefficients in a given iteration were considered selected in that resampling round. Selection frequency was then calculated across all 50 iterations. Metabolites selected in at least 75% of iterations were defined as internally prioritized metabolite features with high selection stability and were retained for downstream contextual analyses. The mean absolute coefficient across resampling iterations was calculated to summarize the relative contribution of each selected feature within the penalized models. LASSO logistic regression was implemented using the scikit-learn package (v1.8.0) in Python (v3.11.11).

This LASSO framework was not designed to establish a validated metabolite-based prediction model. External validation, formal calibration assessment, net reclassification improvement, and comparison against established clinical risk models were not performed. Accordingly, the selected metabolites should be interpreted as internally prioritized features associated with incident hospital-diagnosed migraine in the present dataset, rather than as externally validated biomarkers or a clinically actionable prediction panel.

9. Brain MRI acquisition and metabolite–brain structural association analyses

Brain structural magnetic resonance imaging (MRI) data were obtained from the UKB imaging study. MRI acquisition was performed using a standardized imaging protocol on a Siemens Skyra 3.0-T scanner equipped with a 32-channel head coil. Detailed procedures for image acquisition, quality control, and image processing have been described in the UKB documentation and related publications.[12,13] In brief, T1-weighted structural images and T2-weighted fluid-attenuated inversion recovery (FLAIR) images were processed using established automated pipelines to derive imaging-derived phenotypes (IDPs).[14-16]

The present analyses focused on four global brain structural phenotypes provided by the UKB: white matter hyperintensity (WMH) volume (field 25781), cortical gray matter volume (CGV; field

25001), total gray matter volume (TGV; field 25005), and subcortical gray matter volume (SGV; field 26517). WMH volume, representing the burden of white matter hyperintensities segmented from FLAIR images, was considered a marker of cerebral small vessel disease. Due to its skewed distribution and the presence of zero values, WMH volume was transformed as log(WMH + 1) prior to analysis. Gray matter volume measures were analyzed on their original scales. Total intracranial volume (TIV; field 26521) and assessment centre (field 54) were included as additional covariates to account for head size and site-related variability.

Brain MRI data were acquired during imaging visits occurring after baseline assessment and at varying times relative to migraine onset. Accordingly, the temporal relationship between imaging phenotypes and migraine onset was not uniform across individuals, and metabolite–MRI findings were interpreted as associations rather than directional effects.

Associations between LASSO-prioritized metabolites and brain structural phenotypes were examined using multivariable linear regression, with each metabolite analyzed separately as the independent variable and each MRI phenotype as the dependent variable. All models were adjusted for the baseline covariates described above, in addition to assessment centre and TIV. Metabolites were standardized to z-scores before analysis.

To facilitate comparison across metabolites and outcomes, two types of coefficients were reported. First, metabolite-standardized coefficients were derived from models in which metabolites were standardized but imaging outcomes remained on their original scales, reflecting the absolute change in imaging measures per 1-SD increase in metabolite level. Second, fully standardized coefficients were derived by standardizing both metabolites and imaging phenotypes, allowing direct comparison of association magnitudes across different brain outcomes. Multiple testing was addressed using Bonferroni correction across the 12 LASSO-prioritized metabolites and four brain structural MRI phenotypes.

10. Polygenic risk score analyses

Polygenic risk scores (PRSs) for migraine were constructed using summary-level GWAS data from FinnGen R12 (https://www.finngen.fi/en), comprising 55,116 migraine cases and 445,232 controls.[17] The UKB metabolomics participants used as the target sample in the present study were not included in the FinnGen base GWAS. Therefore, no direct sample overlap between the base GWAS and the target metabolomics sample was expected.

PRSs were generated using PRSice software.[18] Linkage disequilibrium (LD) clumping was applied using an r^2 threshold of 0.1 and a clumping window of 250 kb. Polygenic scores were calculated across several representative GWAS *P*-value thresholds, including $P < 5\times10^{-8}$ (pT_5e-8), $P < 5\times10^{-6}$ (pT_5e-6), $P < 1\times10^{-5}$ (pT_1e-5), $P < 1\times10^{-4}$ (pT_1e-4), and $P < 1\times10^{-3}$ (pT_1e-3).[19]

Associations between migraine PRSs and LASSO-prioritized plasma metabolites were evaluated using multivariable linear regression models. PRSs were treated as continuous predictors, and metabolites were treated as continuous dependent variables. All models were adjusted for the baseline covariates used in the main analyses, with the exception of ethnicity, because PRS analyses were restricted to participants of European ancestry. Multiple testing was addressed using Bonferroni correction across the 12 LASSO-prioritized metabolites and five predefined PRS construction thresholds.

11. Cross-trait overlap analyses and exploratory structural equation modeling

To further contextualize the identified metabolite signatures, we examined their associations with selected sleep-related and affective traits. Sleep duration was assessed using self-reported average hours of sleep per day (field 1160), and insomnia symptoms were derived from baseline questionnaire

responses on sleep difficulties (field 1200). Depression and anxiety were defined using UKB first-occurrence records. Depression was identified from fields 130894 and 130896, corresponding to the dates of first reported depressive episode (F32) and recurrent depressive disorder (F33), respectively. Anxiety was identified from fields 130904 and 130906, corresponding to the dates of first reported phobic anxiety disorders (F40) and other anxiety disorders (F41), respectively. Prevalent status at baseline was defined according to whether the first-occurrence date occurred on or before the baseline assessment date. Associations between LASSO-prioritized plasma metabolites and each sleep-related or affective trait were evaluated using multivariable linear regression models. In these analyses, each trait was modeled separately as the independent variable, and standardized metabolite levels were treated as dependent variables. All models were adjusted for the same baseline covariates used in the primary analyses. For the analysis in which sleep duration was the exposure variable, sleep duration was omitted from the covariate set. Multiple testing was addressed using Bonferroni correction across the 12 LASSO-prioritized metabolites and four selected sleep-related or affective traits.

Exploratory structural equation modeling (SEM) was performed to summarize statistical interrelationships among selected sleep-related or affective traits, a latent metabolite factor, and incident hospital-diagnosed migraine. The SEM analyses were implemented using the lavaan package in R. The latent metabolite factor was specified using the 12 LASSO-prioritized metabolites as indicators. All metabolite indicators were standardized before model fitting.

Four separate SEM models were fitted for depression, anxiety, insomnia, and sleep duration. Depression and anxiety were defined as binary prevalent traits, insomnia was modeled using the baseline insomnia variable, and sleep duration was modeled as a continuous variable. Each exposure variable was standardized before SEM to improve comparability of model coefficients. Incident hospital-diagnosed migraine was coded as a binary outcome and treated as an ordered variable in lavaan. Models were estimated using the weighted least squares mean- and variance-adjusted estimator (WLSMV), with the latent factor variance standardized using std.lv = TRUE.

For each model, the latent metabolite factor was regressed on the exposure trait and baseline covariates, and incident hospital-diagnosed migraine was regressed on both the latent metabolite factor and the exposure trait. The adjusted covariates included age, sex, educational attainment, body mass index, socioeconomic status, ethnicity, smoking status, and alcohol consumption. Categorical covariates were dummy-coded before model fitting. Model-based indirect, direct, and total association parameters were defined as $IE = a \times b$, $DE = c'$, and $TE = c' + a \times b$, respectively, where a represents the association between the exposure trait and the latent metabolite factor, b represents the association between the latent metabolite factor and incident hospital-diagnosed migraine, and c′ represents the direct association between the exposure trait and incident hospital-diagnosed migraine conditional on the latent metabolite factor.

Standardized factor loadings were extracted to characterize the contribution of each LASSO-prioritized metabolite to the latent metabolite factor. Model-based association parameters and standardized coefficients were also extracted. Model fit was evaluated using the comparative fit index (CFI), Tucker–Lewis index (TLI), root mean square error of approximation (RMSEA), and standardized root mean square residual (SRMR). SEM analyses were conducted using the imputed analytic dataset.

These SEM analyses were intended as exploratory integrative models to summarize statistical interrelationships among affective or sleep-related traits, the prioritized metabolite signature, and incident hospital-diagnosed migraine. They should not be interpreted as formal causal mediation analyses, because the exposure traits and metabolites were assessed at or around baseline, temporal

ordering among all variables could not be fully established, bidirectional relationships between migraine and affective or sleep-related traits are possible, and residual confounding cannot be excluded. All regression and SEM analyses were performed in R (v4.2.1).

References

1 Sudlow, C. *et al.* UK biobank: an open access resource for identifying the causes of a wide range of complex diseases of middle and old age. *PLoS Med* **12**, e1001779 (2015). https://doi.org/10.1371/journal.pmed.1001779

2 Elliott, P., Peakman, T. C. & Biobank, U. K. The UK Biobank sample handling and storage protocol for the collection, processing and archiving of human blood and urine. *Int J Epidemiol* **37**, 234-244 (2008). https://doi.org/10.1093/ije/dym276

3 Wurtz, P. *et al.* Quantitative Serum Nuclear Magnetic Resonance Metabolomics in Large-Scale Epidemiology: A Primer on -Omic Technologies. *Am J Epidemiol* **186**, 1084-1096 (2017). https://doi.org/10.1093/aje/kwx016

4 Tzoulaki, I., Ebbels, T. M., Valdes, A., Elliott, P. & Ioannidis, J. P. Design and analysis of metabolomics studies in epidemiologic research: a primer on -omic technologies. *Am J Epidemiol* **180**, 129-139 (2014). https://doi.org/10.1093/aje/kwu143

5 Soininen, P., Kangas, A. J., Wurtz, P., Suna, T. & Ala-Korpela, M. Quantitative serum nuclear magnetic resonance metabolomics in cardiovascular epidemiology and genetics. *Circ Cardiovasc Genet* **8**, 192-206 (2015). https://doi.org/10.1161/CIRCGENETICS.114.000216

6 You, J. *et al.* Mapping the plasma metabolome to human health and disease in 274,241 adults. *Nat Metab* **7**, 2366-2384 (2025). https://doi.org/10.1038/s42255-025-01371-1

7 Zhang, Y. *et al.* Large-scale proteomic analyses of incident Alzheimer's disease reveal new pathophysiological insights and potential therapeutic targets. *Mol Psychiatry* **30**, 2347-2361 (2025). https://doi.org/10.1038/s41380-024-02840-x

8 Ho, D., Imai, K., King, G. & Stuart, E. A. MatchIt: nonparametric preprocessing for parametric causal inference. *Journal of statistical software* **42**, 1-28 (2011).

9 Lehallier, B. *et al.* Undulating changes in human plasma proteome profiles across the lifespan. *Nat Med* **25**, 1843-1850 (2019). https://doi.org/10.1038/s41591-019-0673-2

10 Piehl, N. *et al.* Cerebrospinal fluid immune dysregulation during healthy brain aging and cognitive impairment. *Cell* **185**, 5028-5039 e5013 (2022). https://doi.org/10.1016/j.cell.2022.11.019

11 Tibshirani, R. Regression shrinkage and selection via the lasso. *Journal of the Royal Statistical Society Series B: Statistical Methodology* **58**, 267-288 (1996).

12 Miller, K. L. *et al.* Multimodal population brain imaging in the UK Biobank prospective epidemiological study. *Nat Neurosci* **19**, 1523-1536 (2016). https://doi.org/10.1038/nn.4393

13 Alfaro-Almagro, F. *et al.* Image processing and Quality Control for the first 10,000 brain imaging datasets from UK Biobank. *Neuroimage* **166**, 400-424 (2018). https://doi.org/10.1016/j.neuroimage.2017.10.034

14 Desikan, R. S. *et al.* An automated labeling system for subdividing the human cerebral cortex on MRI scans into gyral based regions of interest. *Neuroimage* **31**, 968-980 (2006). https://doi.org/10.1016/j.neuroimage.2006.01.021

15 Fischl, B. *et al.* Whole brain segmentation: automated labeling of neuroanatomical structures in the human brain. *Neuron* **33**, 341-355 (2002). https://doi.org/10.1016/s0896-

6273(02)00569-x
16 Dale, A. M., Fischl, B. & Sereno, M. I. Cortical surface-based analysis. I. Segmentation and surface reconstruction. *Neuroimage* **9**, 179-194 (1999). https://doi.org/10.1006/nimg.1998.0395
17 Kurki, M. I. *et al.* FinnGen provides genetic insights from a well-phenotyped isolated population. *Nature* **613**, 508-518 (2023). https://doi.org/10.1038/s41586-022-05473-8
18 Choi, S. W. & O'Reilly, P. F. PRSice-2: Polygenic Risk Score software for biobank-scale data. *Gigascience* **8** (2019). https://doi.org/10.1093/gigascience/giz082
19 Choi, S. W., Mak, T. S. & O'Reilly, P. F. Tutorial: a guide to performing polygenic risk score analyses. *Nat Protoc* **15**, 2759-2772 (2020). https://doi.org/10.1038/s41596-020-0353-1
